# Transcendental cinema and psychiatry. The case of Blue Velvet by David Lynch

**DOI:** 10.1192/j.eurpsy.2021.1993

**Published:** 2021-08-13

**Authors:** J. Nowocień, N. Szejko

**Affiliations:** 1 Bioethics, Medical University of Warsaw, Warsaw, Poland; 2 Neurology And Bioethics, Medical University of Warsaw, Warsaw, Poland

**Keywords:** transcendence, cinema, mindfulness, psychiatry

## Abstract

**Introduction:**

Term ‘transcendental cinema’ was first used by Paul Schrader in the context of slow cinema, characterized by long shots, austere camerawork and acting devoid of self-consciousness. This style expresses a spiritual state and comes closer to metaphysic dimension. All these features bring transcendental style closer to philosophy of mindfulness characterized by the practice of purposely bringing one’s attention to experiences occurring in the present moment without judgment, a skill one develops through meditation or other training.

**Objectives:**

The purpose of this project is to demonstrate the connection between transcendental style in cinema and mindfulness. Moreover, we would like to present the cinema as a tool approaching meditation and mindfulness. Particularly, we will use the example of David’s Lynch movie Blue Velvet.

**Methods:**

In our research we use the approach proposed by Paul Schrader and David Lynch to analyze the principles of mindfulness and transcendental cinema in Blue Velvet.

**Results:**

There are a number of presenting positive impact of mindfulness and meditation on mental and physical health of patient not only with neurological or psychological problems. Transcendental cinema is a representation of mindfulness as it teaches paying attention to single stimulus and staying in one thought. Particularly, the combination of meditation music, slow sequences as well as contemplation of human mind and emotional reactions displayed in Blue Velvet is perfect example of transcendental cinema.

**Conclusions:**

We think that transcendental cinema should be treated as a technique of mindfulness used to understand psychological state of health and disease.

**Disclosure:**

No significant relationships.

